# Learning Dynamics in Biophysical Spiking Network Models Are Shaped by KCC2/NKCC1 Cotransporter Stoichiometry

**DOI:** 10.1111/ejn.70596

**Published:** 2026-07-02

**Authors:** Mohamed Ahmed, Antônio‐Carlos Guimarães de Almeida, Antônio Márcio Rodrigues, Damien Depannemaecker

**Affiliations:** ^1^ Laboratório de Neurociência Experimental e Computacional, Departamento de Engenharia de Biossistemas Universidade Federal de São João del‐Rei (UFSJ) São João del‐Rei Brazil; ^2^ Aix Marseille Univ, INSERM, INS, Inst Neurosci Syst Marseille France

**Keywords:** cation‐chloride cotransporters, chloride homeostasis, computational model, KCC2, neural learning, neuronal excitability, NKCC1, spiking neural network, stoichiometry

## Abstract

Neuronal chloride homeostasis, governed by the opposing actions of the KCC2 exporter and NKCC1 importer, plays a critical role in regulating excitability and plasticity. While their individual functions are well characterized, the quantitative impact of their combined dynamics on learning processes remains poorly understood. In this computational study, we systematically varied KCC2 and NKCC1 activity levels within a biologically grounded realistic spiking neural network (RSNN) model to examine their influence on learning performance. Simulations were conducted with 441 combinations of parameters, under two different NKCC1 stoichiometries: the canonical 1 Na^+^:1 K^+^:2 Cl^−^ ratio and a proposed alternative 1 Na^+^:4 K^+^:5 Cl^−^ configuration. Our results demonstrate that successful learning emerges only within a constrained range of KCC2 and NKCC1 activity. Moreover, the shape and boundaries of this functional window are qualitatively altered by the NKCC1 stoichiometry. Under the 1:1:2 configuration, increased KCC2 activity consistently enhanced learning by stabilizing chloride dynamics. In contrast, under the 1:4:5 stoichiometry, the relationship was non‐monotonic, indicating complex, context‐dependent effects of transporter activity on network performance. These findings highlight the critical role of biophysical transporter properties—such as ion coupling ratios—in shaping the computational capabilities of neural circuits. They underscore the importance of incorporating non‐synaptic, ion homeostatic mechanisms into models of learning, particularly when investigating brain development and neurological disorders where chloride regulation is disrupted.

## Graphical abstract

Neuronal chloride homeostasis, governed by KCC2 and NKCC1 cotransporters, constrains convergence to learning in biophysical spiking neural networks. Systematic exploration of transporter activity shows that successful learning emerges only within a restricted parameter regime, whose boundaries and convergence properties critically depend on NKCC1 stoichiometry.

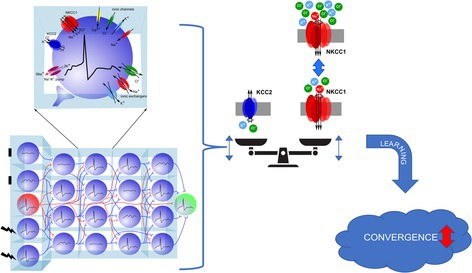

**Abbreviations**
AMPA: α‐amino‐3‐hydroxy‐5‐methyl‐4‐isoxazolepropionic acid receptor
CA1: Cornu Ammonis area 1 (hippocampal subfield)
Cl: chloride ion
DP: developmental period
E/I: excitatory/inhibitory
GABA: gamma‐aminobutyric acid
K^+^
: potassium ion
KCC: potassium–chloride cotransporter
KCC2: potassium–chloride cotransporter 2
LP: learning period
MAD: median absolute deviation
Na^+^
: sodium ion
NKCC: sodium–potassium–chloride cotransporter
NKCC1: sodium–potassium–chloride cotransporter 1
NMDA: *N*‐methyl‐D‐aspartate receptor
*Q*_KCC2_
: maximal flux coefficient for KCC2
*Q*_NKCC1_
: maximal flux coefficient for NKCC1
RSNN: realistic spiking neural network
SEM: standard error of the mean
XOR: exclusive OR

## 1 Introduction

The computational function of neural circuits arises from the interplay between synaptic inputs, intrinsic membrane properties, and cellular mechanisms that regulate neuronal excitability. A fundamental requirement for functional stability in these circuits is the balance between excitatory and inhibitory influences (E/I balance), which ensures both the containment of runaway excitation and the preservation of dynamic range for information processing (Herstel and Wierenga 2021). In the central nervous system (CNS), excitation is primarily mediated by glutamatergic synapses, while inhibition is mediated by γ‐aminobutyric acid (GABA) and glycine (Sears and Hewett 2021; Schwartz 1988; Treiman 2001). Traditionally, this balance has been considered in terms of synaptic strength and connectivity. However, the efficacy of inhibition is also critically shaped by non‐synaptic mechanisms, particularly those governing a neuron's intrinsic excitability and ionic homeostasis.

Inhibitory signaling through ionotropic GABAA and glycine receptors is fundamentally dependent on the intracellular chloride concentration ([Cl^−^]_*i*_), which sets the chloride reversal potential (ECl) and thus determines the direction and magnitude of chloride‐mediated currents (Doyon et al. 2016; Rahmati et al. 2018). The impact of GABAergic inhibition—whether hyperpolarizing, shunting, or depolarizing—is governed by the relationship between ECl and the resting membrane potential (E_m_) (Ben‐Ari et al. 2012; Buzsáki et al. 2007). In mature neurons, low [Cl^−^]_*i*_ (typically ∼5 mM) ensures that E_Cl_ is negative to Em, resulting in robust hyperpolarizing inhibition (Doyon et al. 2016; Schulte et al. 2018). Conversely, elevated [Cl^−^]_*i*_ can render GABAergic currents depolarizing or even excitatory, thereby altering intrinsic excitability and network behavior.

Chloride homeostasis is tightly regulated by the activity of cation‐chloride cotransporters (CCCs), chiefly KCC2, which extrudes chloride, and NKCC1, which imports it (Rivera et al. 1999; Hübner et al. 2001; Russell 2000a; Blaesse et al. 2009). These transporters are essential non‐synaptic regulators of inhibitory efficacy and excitability, operating in parallel to classical synaptic plasticity mechanisms. Through their influence on [Cl^−^]_*i*_, they dynamically modulate the inhibitory landscape of neural circuits, particularly during development or in response to prolonged changes in activity. Importantly, these transporters operate with fixed ion stoichiometries—typically 1 Na^+^:1 K^+^:2 Cl^−^ for NKCC1 and 1 K^+^:1 Cl^−^ for KCC2—though alternative stoichiometries for NKCC1 have been proposed based on thermodynamic and experimental evidence (Brumback and Staley 2008).

The expression and functional contribution of CCCs change dynamically across development. During early postnatal stages, NKCC1 expression dominates, leading to elevated [Cl^−^]_*i*_ and depolarizing GABAergic responses, which are thought to promote neurogenesis, migration, and synaptogenesis (Peerboom and Wierenga 2021; Fiumelli et al. 2013; Tang et al. 2021; Nakanishi et al. 2007; Young et al. 2012). As development progresses, KCC2 expression increases, driving the canonical “GABA shift”—a transition from depolarizing to hyperpolarizing GABAergic transmission that supports the emergence of mature inhibitory control (Rivera et al. 1999; Ben‐Ari et al. 2007; Kahle, Staley, Nahed, Barrett, et al. 2008).

Disruptions in this tightly regulated CCC expression profile are implicated in a range of neurodevelopmental, neurological, and psychiatric disorders characterized by altered excitability and impaired cognition (Kahle, Staley, Nahed, Gamba, et al. 2008; Cherubini et al. 2022). For example, increased NKCC1 expression and reduced KCC2 function have been linked to pathological depolarizing inhibition and learning deficits in models of autism spectrum disorder and Down syndrome, both of which are ameliorated by NKCC1 blockade (Deidda et al. 2015; Tyzio et al. 2014). In rodent models of schizophrenia, CCC dysregulation correlates with cognitive impairments that can be reversed through bumetanide treatment, further underscoring the link between chloride regulation and higher order function (Kim et al. 2021).

Multiple experimental works confirm that CCC activity directly influences learning and memory, even in the absence of disease. Genetic or pharmacological reduction of KCC2 in adult rodents impairs spatial learning, alters network oscillations, and increases seizure susceptibility (Simonnet et al. 2023; Tornberg et al. 2005; Moore et al. 2019). Conversely, inhibition of NKCC1 improves learning in both healthy and disease models (Deidda et al. 2015; Dargaei et al. 2018), while KCC2 overexpression promotes dendritic spine maturation and synaptic plasticity (Nakamura et al. 2019; Kasai et al. 2010). These findings collectively position CCCs as key modulators of not just inhibitory tone, but also of network‐level cognitive performance.

Computational modeling has increasingly been employed to elucidate the impact of chloride dynamics on neural computation. Biophysical simulations have demonstrated how disrupted CCC function can destabilize E/I balance and promote epileptiform activity (Buchin et al. 2016; Lopes et al. 2013), while single‐neuron models have shown how changes in [Cl^−^]_*i*_ affect input–output transformations and spike threshold modulation (Currin et al. 2020; Currin and Raimondo 2022). At the molecular level, thermodynamic models have estimated transporter energetics and ion‐coupling constraints, highlighting the importance of stoichiometry in determining net ion flow and chloride accumulation (Brumback and Staley 2008; Kolbaev et al. 2020). Despite this progress, the quantitative relationship between graded CCC activity, ion transporter stoichiometry, and network‐level learning remains poorly understood. In particular, it is not known whether there exists a bounded functional regime of KCC2 and NKCC1 activity that optimally supports learning, nor how this regime is modulated by fundamental biophysical constraints such as the ion‐coupling stoichiometry of NKCC1.

To address this gap, we employed a biophysical spiking neural network model (RSNN) to systematically investigate how variations in KCC2 and NKCC1 activity influence learning performance. We simulated a learning task across a two‐dimensional activity space (21 × 21 grid), under two distinct NKCC1 stoichiometries: the canonical 1 Na^+^:1 K^+^:2 Cl^−^ configuration and a proposed alternative 1 Na^+^:4 K^+^:5 Cl^−^ model. We hypothesized that (1) learning efficacy would be restricted to a specific functional window of CCC activity, and (2) the structure of this window would be qualitatively altered by the transporter's underlying ion stoichiometry. Our findings reveal that effective learning is confined to a sharply bounded region of CCC activity space and that this region's topology is qualitatively modulated by NKCC1 stoichiometry. These results highlight how biophysical constraints at the molecular level shape emergent cognitive function at the network level and underscore the importance of modeling non‐synaptic ionic mechanisms in theories of learning and brain disease.

## 2 Methods

### 2.1 Neural Network Model

The computational model used in this study is a modified version of the biophysically detailed spiking neural network model (RSNN) previously developed by our group (Depannemaecker et al. 2020; Rodrigues et al. 2021). The RSNN simulates a small hippocampal‐like cell assembly comprising 23 excitatory pyramidal neurons and one inhibitory basket cell interneuron. This reduced configuration was chosen to isolate the mechanistic role of chloride homeostasis in learning, rather than to reproduce the full statistical composition of a biological hippocampal network. In the hippocampus, inhibitory interneurons typically represent a minority of the neuronal population (on the order of 10%–15%, depending on subfield and classification criteria) yet exert a disproportionately strong influence on network excitability through fast perisomatic inhibition. In the present model, the single inhibitory neuron should therefore be interpreted as an effective population unit, representing the aggregate inhibitory influence of a local interneuron ensemble (e.g., basket cells) onto the excitatory assembly. This formulation preserves the essential feedback inhibition motif while maintaining computational tractability, which is critical given the explicit modeling of ionic electrodiffusion, dynamic volume changes, and cation–chloride cotransporter kinetics. Importantly, this minimal architecture allows systematic exploration of a large parameter space, as it is described below, under controlled initial conditions, enabling the identification of learning regimes shaped by transporter balance and stoichiometry.

A key feature of the model is its integration of multiple non‐synaptic mechanisms, including ionic electrodiffusion, dynamic intracellular and extracellular volume changes, and active ion pumping via Na^+^/K^+^‐ATPase. The membrane potential (*V*_*m*_) of each neuron is calculated using the Goldman‐Hodgkin‐Katz (GHK) equation, with ion channel dynamics governed by the Hodgkin‐Huxley formalism. The equations are similar as in previous work (Depannemaecker et al. 2020).

For the present study, several modifications were implemented. First, the network's feed‐forward architecture was augmented with the inclusion of recurrent excitatory synapses from the pyramidal neurons back to the basket cell interneuron, establishing a more robust feedback inhibition loop. Second, to stabilize the learning process, the strength of all synapses was constrained by a saturation threshold. Third, to isolate the effects of the CCC activity scalers, all simulations were initiated from identical conditions. All synaptic weights were uniformly initialized to a value of 0.3, and the initial membrane potential was set to the same value for all neurons in the network.

### 2.2 KCC2 and NKCC1 Modeling

For this study, the simplified cotransporter models from the original RSNN framework (Depannemaecker et al. 2020) were replaced with more detailed models based on the work of (Rodrigues et al. 2021). These models incorporate complex ionic affinity terms, providing a more realistic representation of transporter function.

The total ionic flux for each cotransporter (Φ_*total,CCC*_) was calculated as follows:

(1) $$\Phi_{\text{total},\mathrm{CCC}}=Q_{\mathrm{CCC}}\phi_{\mathrm{CCC}}f\left(t\right),$$

where the time‐dependent modulation term is defined as follows:

(2) $$f\left(t\right)=S_{\mathrm{CCC}}f_{\mathrm{CCC}}\left(t\right).$$

Here, *S*_*CCC*_ is a dimensionless activity scaler (varied independently from 0 to 20 for KCC2 and NKCC1), *Q*_*CCC*_ is the maximal flux coefficient in mmol/(cm^2^·s), *ϕ*_*CCC*_ is a dimensionless biophysical transport term, and *f*_*CCC*_(*t*) is the piecewise‐linear temporal profile described in Equation (6). Because every simulation starts from identical physiological states, the only variables that differentiate one run from another are the temporal profile *f*_*CCC*_(*t*) and its scaled form *f*(*t*), which together modulate KCC2/NKCC1 flux. Plotting the single composite curve *f*(*t*) therefore displays, in one trace, the complete set of initial‐condition variations driving all results.

Two distinct sets of simulations were conducted, one for each NKCC1 stoichiometry. The maximal flux factors (*Q*) were tuned independently for each stoichiometric condition. This was a necessary methodological step to establish a comparable and physiologically relevant dynamic range for analysis, ensuring that the full spectrum of network behaviors—from hypo‐excitable to hyper‐excitable—could be captured within the tested parameter space for both the potent 1:4:5 model and the canonical 1:1:2 model.

The inclusion of an alternative NKCC1 stoichiometry (1 Na^+^:4 K^+^:5 Cl^−^) is motivated by thermodynamic and experimental considerations suggesting that the canonical 1 Na^+^:1 K^+^:2 Cl^−^ coupling may not fully capture the operational constraints of NKCC1 under all physiological conditions. Thermodynamic analyses and flux measurements have shown that, particularly in immature neurons and in regimes of high intracellular sodium and potassium gradients, a higher effective chloride coupling may be required to account for the magnitude and direction of observed chloride accumulation (Brumback and Staley 2008; Kaila et al. 2014). Although direct experimental quantification of NKCC1 stoichiometry in intact neurons remains challenging, several studies have proposed that the transporter can operate with variable effective coupling ratios depending on ionic gradients, membrane potential, and regulatory state. In the present study, the 1 Na^+^:4 K^+^:5 Cl^−^ configuration is therefore not introduced as a definitive molecular description, but as a theoretically grounded alternative that allows us to test the sensitivity of network‐level learning dynamics to fundamental biophysical constraints imposed by transporter coupling. By comparing learning landscapes obtained under the canonical and alternative stoichiometries, we explicitly assess whether the qualitative conclusions regarding chloride‐dependent learning are robust or whether they depend critically on assumptions about NKCC1 ion coupling.

#### 2.2.1 Simulations With 1:4:5 NKCC1 Stoichiometry

For this set of simulations, the maximal flux factors were set to *Q*_*KCC*2_ = 1.1928 × 10^−7^ mmol/(m^2^·s) and *Q*_*NKCC*1_ = −1.775 × 10^−7^ mmol/(m^2^·s). The flux dynamics (*ϕ*) were governed by

(3) $$\begin{array}{c} \phi_{\mathrm{KCC}2}=\frac{{\left[K^{+}\right]}_{i}{\left[{\mathrm{Cl}}^{-}\right]}_{i}}{\left({\left[K^{+}\right]}_{i}+40.0\left(1+\frac{{\left[K^{+}\right]}_{o}}{7.39}\right)\right)\left({\left[{\mathrm{Cl}}^{-}\right]}_{i}+14.5\left(1+\frac{{\left[{\mathrm{Cl}}^{-}\right]}_{o}}{91.02}\right)\right)}\\ -\frac{{\left[K^{+}\right]}_{o}{\left[{\mathrm{Cl}}^{-}\right]}_{o}}{\left({\left[K^{+}\right]}_{o}+7.39\left(1+\frac{{\left[K^{+}\right]}_{i}}{40.0}\right)\right)\left({\left[{\mathrm{Cl}}^{-}\right]}_{o}+91.02\left(1+\frac{{\left[{\mathrm{Cl}}^{-}\right]}_{i}}{14.5}\right)\right)}, \end{array}$$

(4) $$\begin{array}{c} \phi_{\text{NKCC}1,1:4:5}=\frac{{\left[{\mathrm{Na}}^{+}\right]}_{o}{\left({\left[K^{+}\right]}_{o}\right)}^{4}{\left({\left[{\mathrm{Cl}}^{-}\right]}_{o}\right)}^{5}}{\left({\left[{\mathrm{Na}}^{+}\right]}_{o}+70\left(1+\frac{{\left[{\mathrm{Na}}^{+}\right]}_{i}}{6}\right)\right){\left({\left[K^{+}\right]}_{o}+2.3\left(1+\frac{{\left[K^{+}\right]}_{i}}{68}\right)\right)}^{4}{\left({\left[{\mathrm{Cl}}^{-}\right]}_{o}+25\left(1+\frac{{\left[{\mathrm{Cl}}^{-}\right]}_{i}}{7}\right)\right)}^{5}}\\ -\frac{{\left[{\mathrm{Na}}^{+}\right]}_{i}{\left({\left[K^{+}\right]}_{i}\right)}^{4}{\left({\left[{\mathrm{Cl}}^{-}\right]}_{i}\right)}^{5}}{\left({\left[{\mathrm{Na}}^{+}\right]}_{i}+6\left(1+\frac{{\left[{\mathrm{Na}}^{+}\right]}_{o}}{70}\right)\right){\left({\left[K^{+}\right]}_{i}+2.3\left(1+\frac{{\left[K^{+}\right]}_{o}}{2.3}\right)\right)}^{4}{\left({\left[{\mathrm{Cl}}^{-}\right]}_{i}+7\left(1+\frac{{\left[{\mathrm{Cl}}^{-}\right]}_{o}}{25}\right)\right)}^{5}}, \end{array}$$

#### 2.2.2 Simulations With 1:1:2 NKCC1 Stoichiometry

For this set of simulations, the maximal flux factors were experimentally tuned to *Q*_*KCC*2_ = 1.1928 × 10^−8^ mmol/(m^2^·s) and *Q*_*NKCC*1_ = 2.414 × 10^−8^ mmol/(m^2^·s). This adjustment of the transporter capacities was a necessary methodological step, as preliminary simulations revealed that without this tuning, the system was insensitive to the activity scalers. The biophysical model for KCC2 (*ϕ*_*KCC*2_) was identical to that used in the 1:4:5 simulations. The canonical 1 Na^+^:1 K^+^:2 Cl^−^ NKCC1 model was defined as

(5) $$\begin{array}{c} \phi_{\text{NKCC}1,1:1:2}=\frac{{\left[{\mathrm{Na}}^{+}\right]}_{o}{\left[K^{+}\right]}_{o}{\left({\left[{\mathrm{Cl}}^{-}\right]}_{o}\right)}^{2}}{\left({\left[{\mathrm{Na}}^{+}\right]}_{o}+70\left(1+\frac{{\left[{\mathrm{Na}}^{+}\right]}_{i}}{6}\right)\right)\left({\left[K^{+}\right]}_{o}+2.3\left(1+\frac{{\left[K^{+}\right]}_{i}}{68}\right)\right){\left({\left[{\mathrm{Cl}}^{-}\right]}_{o}+25\left(1+\frac{{\left[{\mathrm{Cl}}^{-}\right]}_{i}}{7}\right)\right)}^{2}}\\ -\frac{{\left[{\mathrm{Na}}^{+}\right]}_{i}{\left[K^{+}\right]}_{i}{\left({\left[{\mathrm{Cl}}^{-}\right]}_{i}\right)}^{2}}{\left({\left[{\mathrm{Na}}^{+}\right]}_{i}+6\left(1+\frac{{\left[{\mathrm{Na}}^{+}\right]}_{o}}{70}\right)\right)\left({\left[K^{+}\right]}_{i}+2.3\left(1+\frac{{\left[K^{+}\right]}_{o}}{2.3}\right)\right){\left({\left[{\mathrm{Cl}}^{-}\right]}_{i}+7\left(1+\frac{{\left[{\mathrm{Cl}}^{-}\right]}_{o}}{25}\right)\right)}^{2}}, \end{array}$$

where [*X*]_*i*_ and [*X*]_*o*_ represent the intracellular and extracellular concentrations of ion *X*, respectively.

#### 2.2.3 Cotransporters Parameter Estimation

Estimates of maximal KCC‐mediated ionic fluxes in the range of 10^−8^–10^−6^ mmol/(cm^2^·s) are supported by Rb^+^ influx measurements (Payne 1997; Gillen and Forbush 1999), by experimentally observed chloride recovery timescales (Kaila et al. 2014; Rivera et al. 1999), and by quantitative models of neuronal chloride homeostasis (Doyon et al. 2011; Rodrigues et al. 2015). Similarly, experimental Rb^+^ influx data and modeling studies indicate that maximal NKCC‐mediated ionic fluxes typically lie in the range of 10^−6^–10^−4^ mmol/(cm^2^·s) (Doyon et al. 2011; Rodrigues et al. 2015; Haas and Forbush 1986; Tas et al. 1987; Russell 2000b). These orders of magnitude were therefore adopted as physiologically grounded constraints for the maximal transport rates used to adjust the kinetic parameters of both cotransporters. In this formulation, the maximal flux constants represent effective transport capacity, capturing the combined effects of transporter expression level and intrinsic molecular activity rather than absolute protein abundance.

**FIGURE 1:**
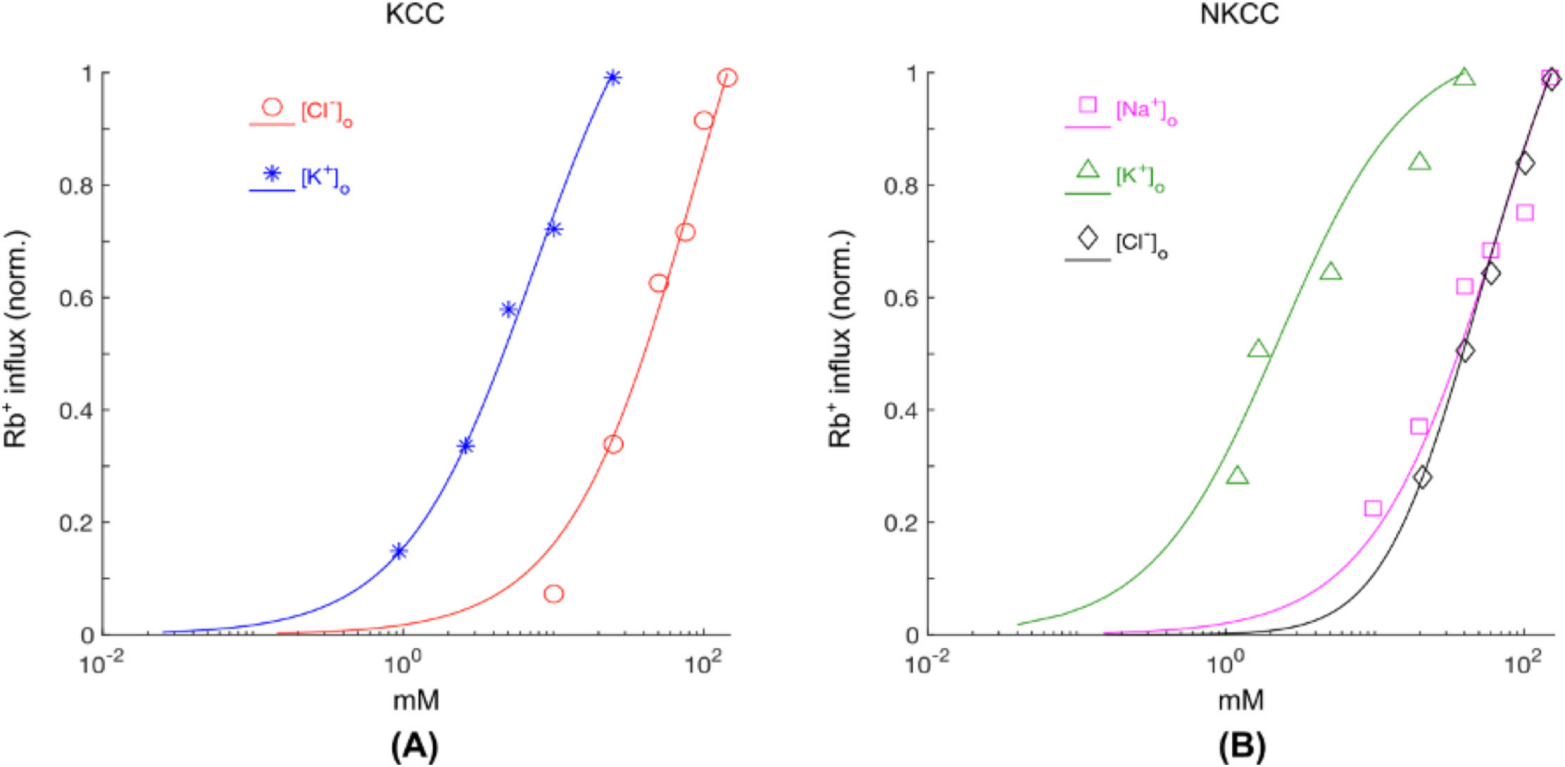
Adjustment of extracellular dissociation constants for KCC and NKCC cotransporters. (A) KCC cotransporter activity as a function of extracellular potassium 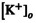 and chloride 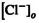 concentrations. Rb^+^ influx is shown normalized to its value at 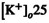 mM (blue symbols) and 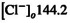 mM (red symbols). Circles and asterisks correspond to experimental measurements reported by [C]. Solid lines represent model simulations using Equation (3), which describes ionic fluxes mediated by KCC. The extracellular dissociation constants for K^+^ and Cl^−^ were adjusted to reproduce the experimentally observed dependence of transport activity. (B) NKCC cotransporter activity as a function of extracellular sodium 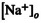, potassium 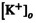, and chloride 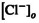 concentrations. Rb^+^ influx is normalized to its value at 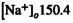 mM (magenta), 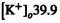 mM (green), and 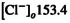 mM (black). Triangles, squares, and diamonds indicate experimental data from [I = 47]. Solid lines correspond to model simulations based on Equation (4) for NKCC‐mediated ionic influx, with extracellular dissociation constants for Na^+^, K^+^, and Cl^−^ adjusted to match the experimental transport profiles.

With respect to ion affinities, the dissociation constants of both cotransporters were constrained using experimental transport data and adjusted separately for the extracellular and intracellular faces of the membrane. On the extracellular side, dissociation constants for each permeant ion were tuned to reproduce the experimentally reported dependence of normalized Rb^+^ influx on external ionic concentrations, as illustrated in Figure 1 for KCC and NKCC. Specifically, the extracellular affinities were adjusted so that the model accurately captures the saturation profiles and relative sensitivities to 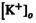, 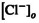, and, in the case of NKCC, 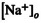, without enforcing absolute flux magnitudes. In contrast, dissociation constants at the intracellular face were selected based on thermodynamic considerations, imposing that both cotransporters operate close to electrochemical equilibrium under resting conditions, while preserving their characteristic transport directionality—namely, net Cl^−^ efflux for KCC and net Cl^−^ influx for NKCC. This strategy ensures consistency with experimentally observed steady‐state chloride homeostasis, while allowing transporter activity to deviate from equilibrium under perturbed conditions.

#### 2.2.4 Temporal Modulation

To emulate the expression of KCC2 and NKCC1 during the developmental period (DP) (Rivera et al. 1999; Kahle, Staley, Nahed, Barrett, et al. 2008), we defined a temporal modulation profile, *f*_*CCC*_(*t*). This profile acts as a step function, updated at the beginning of each epoch, that modulates the developmental rise of KCC2 and NKCC1 during the first 20 epochs (the DP) and remains constant thereafter. The value of the function at any given epoch *t* is determined by a piecewise linear equation:

(6) $$f_{\mathrm{CCC}}\left(t\right)=\left\{\begin{array}{c} m_{1,\mathrm{CCC}}t,\;\text{if}\;1\leq t\leq t_{1}\\ m_{1,\mathrm{CCC}}t_{1}+m_{2,\mathrm{CCC}}\left(t-t_{1}\right),\;\text{if}\;t_{1}<t\leq t_{2}\\ m_{1,\mathrm{CCC}}t_{1}+m_{2,\mathrm{CCC}}\left(t_{2}-t_{1}\right),\;\text{if}\;t_{2}<t \end{array}\right.$$

with breakpoints at *t*_1_ = 10 and *t*_2_ = 20 epochs. We set the slopes to

$$m_{1,\mathrm{KCC}2}=1,m_{2,\mathrm{KCC}2}=3;m_{1,\text{NKCC}1}=2,m_{2,\text{NKCC}1}=2.$$

This yielded transporter‐specific temporal profiles during the DP: KCC2 undergoes a two‐phase ramp with accelerated growth in the late DP, while NKCC1 follows a uniform linear increase. From epoch 21 onward, *f*_*CCC*_(*t*) remains fixed at the value reached at *t* = 20. For the base case where scalers are 1, this value is 40 a.u.

### 2.3 Stimulus Protocol

Input to the network was provided by applying a current injection (I = 300 pA) to the first layer of pyramidal neurons, scaled by a binary stimulus array. Each experimental phase consisted of epochs where a set of four distinct stimulus patterns was presented. Two sets of stimuli were used, corresponding to the two main phases of the simulation:

1. Random Stimulus: Used during the initial DP, this set consisted of four randomly generated binary patterns designed to drive spontaneous‐like activity. These arrays were generated with the constraint that the sum of “1” entries in any row or column could not exceed 2, keeping the overall input intensity relatively low and unstructured.
2. XOR Task Stimulus: Used during the subsequent learning period (LP), this structured set of four binary patterns defines a nonlinear separation task. The XOR task was chosen as a canonical benchmark for nonlinear computation (Depannemaecker et al. 2020), providing a clear and well‐defined measure of learning capacity that is computationally tractable for our biophysically detailed RSNN. The input stimulus matrix is
$${\text{Stimulus}}_{\mathrm{XOR}}=\left[\begin{array}{c} 1\\ 0\\ 0\\ 1 \end{array}\;\begin{array}{c} 1\\ 0\\ 0\\ 1 \end{array}\;\begin{array}{c} 0\\ 1\\ 0\\ 1 \end{array}\;\begin{array}{c} 0\\ 1\\ 0\\ 1 \end{array}\right]$$

where each row represents one of the four patterns presented to the input layer within an epoch.

### 2.4 Learning Rule and Task Definition

The network was trained using an error‐driven synaptic modification rule based on Hebbian and anti‐Hebbian principles, adapted from our previous work (Depannemaecker et al. 2020). Following the presentation of each stimulus pattern, the output neuron's spiking response was compared to a target binary output to generate an error signal (error ∈ {−1, 0, +1}), which directed synaptic modifications.

The target output vector depended on the simulation phase. During the DP, while the network was presented with the four random stimulus patterns, the target output vector was held fixed at [1, 1, 0, 0]. This design ensures that the learning rule remains active, allowing for activity‐dependent synaptic modification that promotes general pathway efficacy without encoding a specific, meaningful map from the random inputs. This prevents premature convergence and avoids the formation of structural bias before the formal learning task begins. During the subsequent LP, the target vector correctly corresponded to the four XOR stimulus patterns, which is also [1, 1, 0, 0].

The randomized feedback matrix (B1) from the original formulation (Depannemaecker et al. 2020) was omitted to isolate the effects of the CCC scalers. The primary performance metric, “epochs to convergence,” was defined as the number of epochs elapsed within the LP until the network first achieved—and subsequently maintained—error‐free classification of all four XOR patterns. An early stopping mechanism was implemented to terminate simulations that did not converge within the 20‐epoch limit of the LP.

Learning convergence was defined using a strict, binary performance criterion based on the network's classification accuracy during the LP. After each epoch, the spiking output of the readout neuron was evaluated for all four XOR input patterns and compared to the corresponding target outputs. An epoch was considered error‐free when the network produced the correct binary response for all four patterns, yielding a total classification error of zero. Convergence was declared at the first epoch in which this zero‐error condition was achieved and subsequently maintained for the remainder of the LP. If the network failed to reach and sustain error‐free performance within the 20‐epoch limit of the LP, the simulation was classified as non‐convergent. This criterion was applied identically across all simulations and parameter combinations, ensuring a consistent and unambiguous definition of learning success.

### 2.5 Simulation Protocol

**FIGURE 2:**
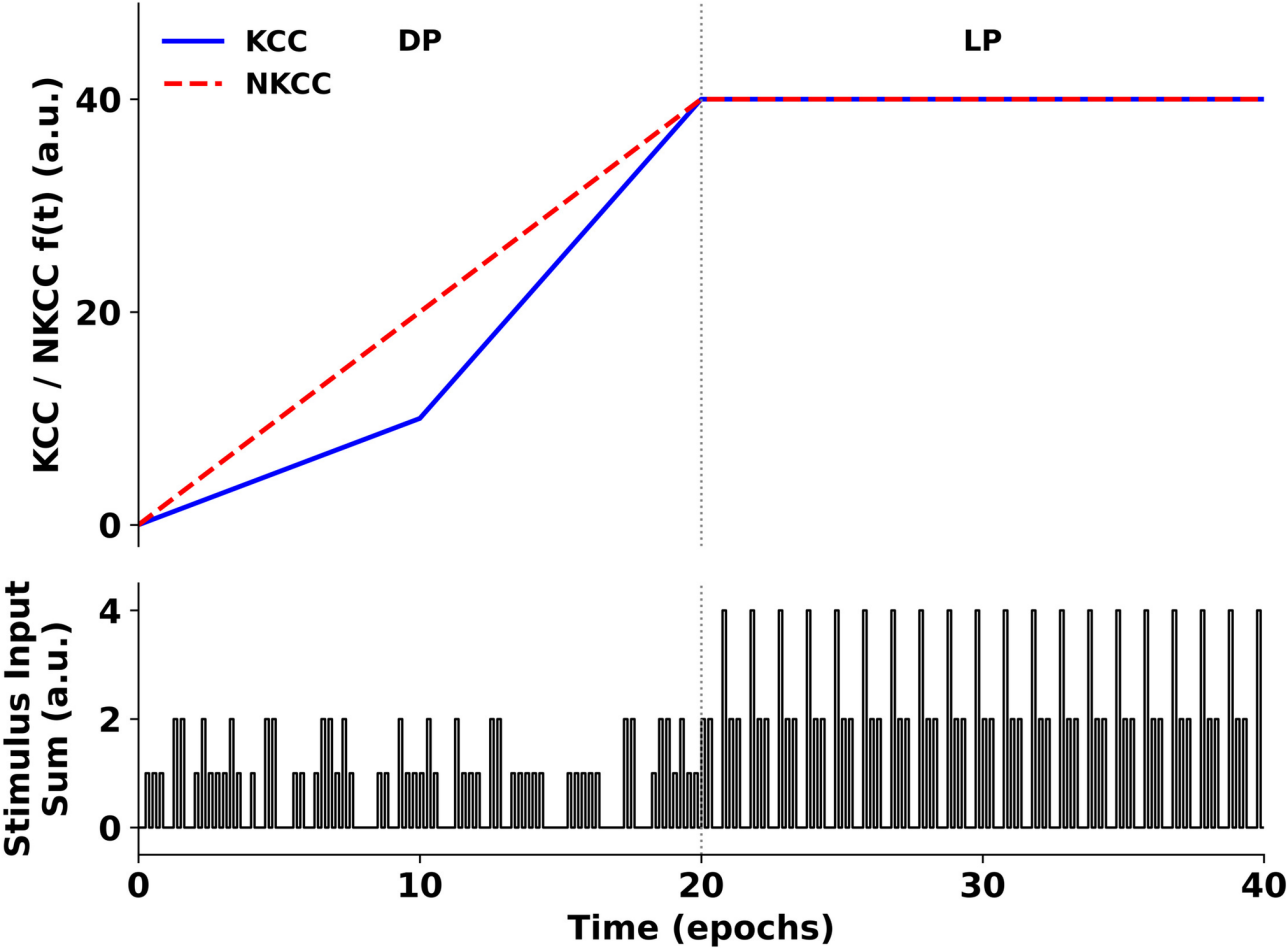
Schematic of the temporal protocol for a single simulation run. The modulation profiles for KCC2 and NKCC1 during the developmental period (DP) are designed to mimic heir known expression trajectory during the perinatal period (Kahle, Staley, Nahed, Barrett, et al. 2008). During the learning period (LP), the value of the modulation function, *f*(*t*), is held constant at the level reached at the end of the DP. This figure illustrates the base case where CCCs activity scalers *S*_*KCC*2_ = 1 and *S*_*NKCC*1_ = 1. The bottom panel shows the corresponding switch from a random stimulus during the DP to the structured XOR task stimulus during the LP.

The simulations performed in this study followed the two‐phase protocol illustrated in Figure 2.

- DP: Lasting 20 epochs, this phase simulated early network development. The network was driven by the random stimulus, and the maximal potential flux of each CCC was dynamically modulated according to the temporal profile *f*(*t*) (Equation 6).
- LP: Immediately following the DP, this phase also lasted 20 epochs. To assess learning in a stable developmental state, the temporal modulation function, *f*(*t*), was held constant at the value it reached at the end of the DP (*t* = 20 epochs) for the remainder of the simulation.

This entire protocol was repeated for each point on a 21 × 21 parameter grid, where *S*_*KCC*2_ and *S*_*NKCC*1_ were varied independently from 0 to 20.

### 2.6 Robustness Analysis

Learning performance was quantified using discrete convergence‐based metrics. For each simulation, we recorded the number of epochs required to reach sustained zero‐error classification during the LP, defined as correct binary responses for all four XOR patterns within an epoch. This epochs‐to‐convergence measure was used as the primary indicator of learning success. Across repeated simulations, robustness was assessed by computing the median epochs‐to‐convergence and the corresponding median absolute deviation (MAD) for each $\left(S_{\mathrm{KCC}2},S_{\text{NKCC}1}\right)$ parameter pair.

In this framework, learning is treated as a binary classification task with a strict success criterion. Network configurations that fail to converge typically exhibit persistent classification errors throughout the LP, rather than a gradual reduction in error magnitude. Under these conditions, continuous error‐based measures—such as cumulative or epoch‐averaged error—are expected to covary with the discrete convergence metric and were therefore not considered separately. The use of a binary convergence criterion ensures a consistent and unambiguous definition of learning success across all simulations and parameter combinations.

To test whether the observed learning landscapes were dependent on a single, specific starting state, we performed a robustness analysis for both NKCC1 stoichiometries. The entire 21 × 21 grid simulation was repeated 10 times. For each of these 10 repetitions, two sources of variability were introduced at network initialization: the intrinsic neural excitability perturbation and random initialization of the synaptic weights of the network.

### 2.7 Software and Implementation

The simulation code was implemented in Fortran 90 and executed on a Linux‐based system (Ubuntu). MATLAB and Python were employed for data extraction, preprocessing, visualization, and analysis. Bash scripts were used to manage parallel execution across simulation batches. Computational resources were provided by the LANEC laboratory, including an SGI cluster with 80 processing cores.

## 3 Results

**FIGURE 3:**
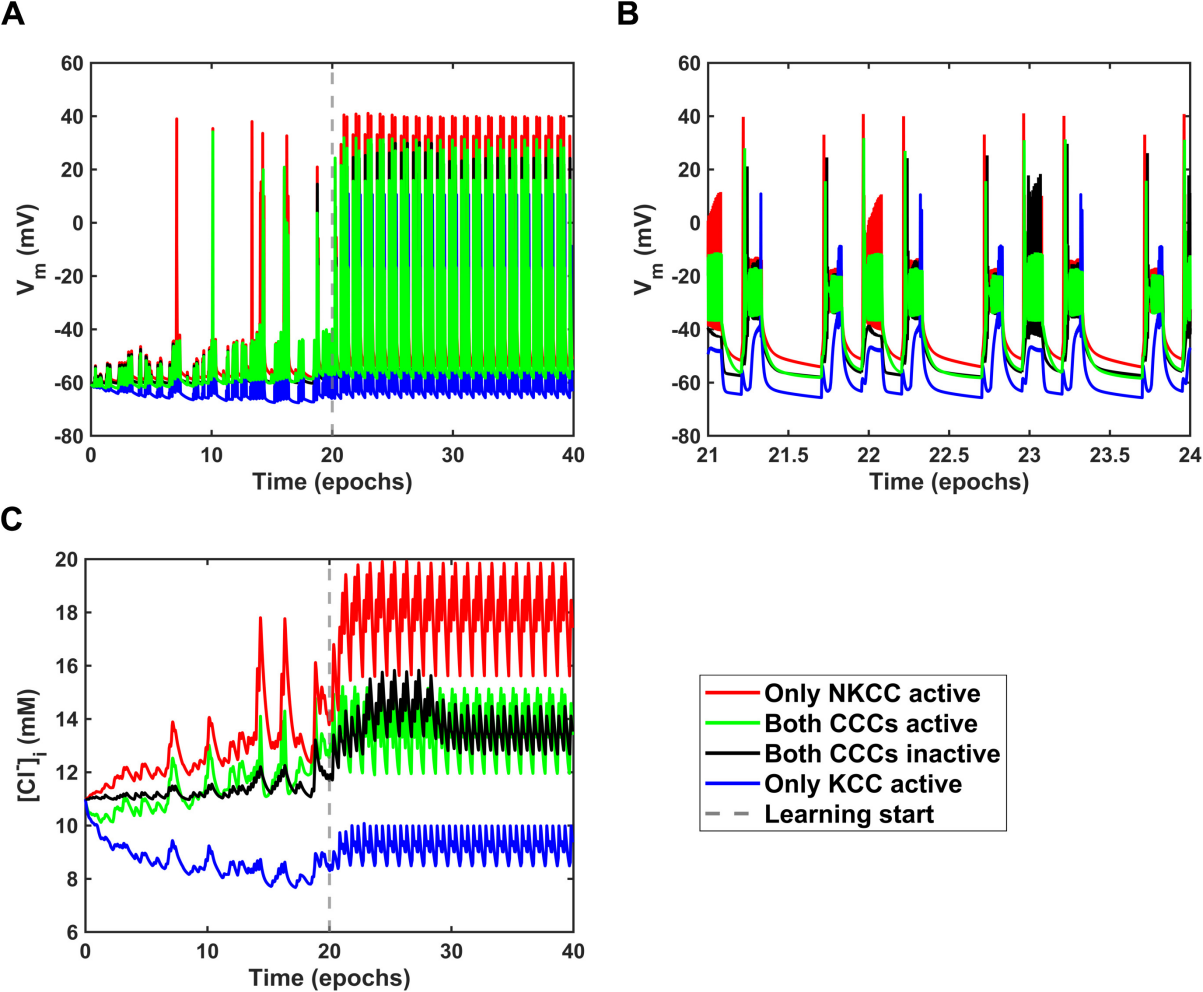
Cation‐chloride cotransporter activity directly modulates intracellular chloride and neuronal excitability. (A) Overview of the membrane potential (*V*_*m*_) for a representative neuron across the 40‐epoch simulation. The four conditions, representing the binary combinations of active/inactive transporters, diverge from a common starting point to establish distinct baseline potentials. (B) A detailed view of epochs 21–24 shows the fine structure of the firing bursts during the learning task, highlighting the computational consequences of the different excitability states. (C) The corresponding overview of intracellular chloride concentration ([Cl^−^]_*i*_) demonstrates that the changes in *V*_*m*_ are driven by the differential homeostatic regulation of chloride. For these illustrative simulations, “active” states correspond to a transporter activity scalers (*S*_*KCC*2_ and *S*_*NKCC*1_) of 3 and 6, respectively, while “inactive” corresponds to a scaler of 0.

To illustrate the direct influence of cotransporter activity on neuronal dynamics, we first simulated four representative baseline conditions corresponding to different combinations of KCC2 and NKCC1 activity. These included a condition where only NKCC1 was active (*S*_*NKCC*1_ = 6.0, *S*_*KCC*2_ = 0), a condition where both transporters were active (*S*_*NKCC*1_ = 6.0, *S*_*KCC*2_ = 3.0), a fully inactive condition (*S*_*NKCC*1_ = 0, *S*_*KCC*2_ = 0), and a condition with only KCC2 active (*S*_*NKCC*1_ = 0, *S*_*KCC*2_ = 3.0). As shown in Figure 3, each configuration produced distinct steady‐state levels of intracellular chloride concentration ([Cl^−^]_*i*_; Panel C), which in turn modulated the resting membrane potential and spike amplitude. These differences were observable both across the entire simulation (Panel A) and during the LP (Panel B), confirming that cotransporter activity exerts a direct, non‐synaptic control over key excitability parameters.

Having established the biophysical plausibility of the model in reproducing these chloride‐dependent excitability shifts, we next examined how they influence learning performance across a broad parameter space. To this end, we systematically varied the activity scalers *S*_*KCC*2_ and *S*_*NKCC*1_ across a 21 × 21 grid, with values ranging from 0 to 20. This exploration was conducted for two distinct NKCC1 stoichiometries: the canonical 1 Na^+^:1 K+:2 Cl^−^ stoichiometry (referred to as 1:1:2) and an alternative 1 Na^+^:4 K^+^:5 Cl^−^ stoichiometry (1:4:5).

**FIGURE 4:**
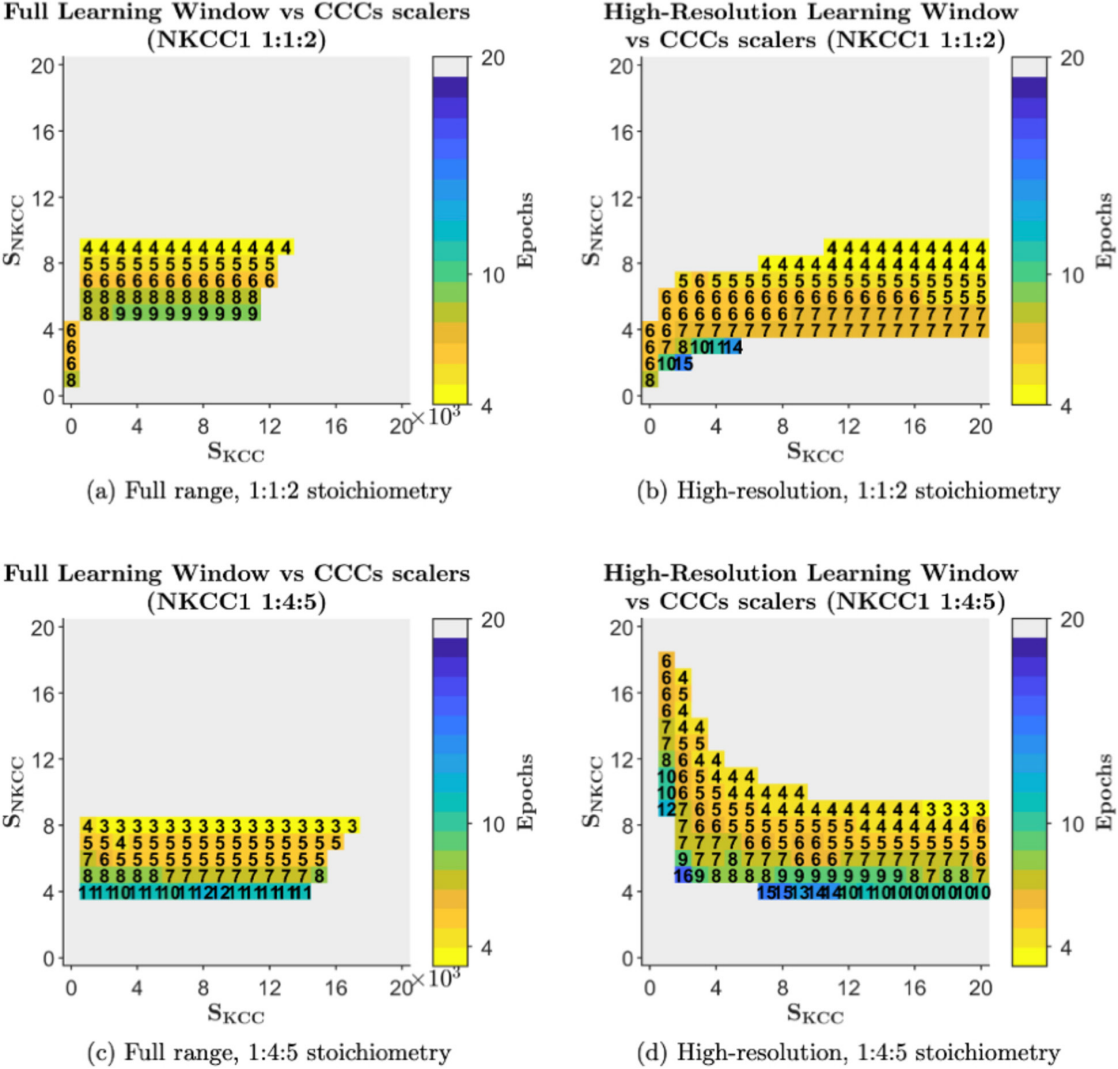
Comparison of learning heatmaps (number of epochs) for two NKCC1 stoichiometries. Panels (A) and (C) show the outcomes across the full tested parameter space for the 1:1:2 and 1:4:5 stoichiometries, respectively. Panels (B) and (D) provide higher resolution views of the optimal learning regions. Heatmap values represent the number of epochs elapsed from the start of the learning period until the network first achieved sustained zero‐error classification across all four XOR patterns. Regions where no such epoch occurred within the 20‐epoch limit are shown as non‐convergent.

The results shown in Figure 4 show that successful learning—defined as convergence within 20 epochs—only occurred within a specific functional window of KCC2 and NKCC1 activity. Outside of this window, networks typically failed to learn due to either insufficient excitability or excessive, destabilizing levels of excitability. Notably, the properties of this functional window were strongly shaped by the stoichiometry of NKCC1. Under the 1:4:5 model, increasing KCC2 activity progressively narrowed the effective range of NKCC1 activity that supported learning (Figure 4B). In contrast, in the 1:1:2 model, the learning‐competent range initially expanded as KCC2 activity increased (Figure 4D). This stoichiometry‐dependent effect was particularly striking under conditions of complete KCC2 blockade: While a narrow range of NKCC1 activity still permitted learning in the 1:1:2 model, learning was entirely abolished in the 1:4:5 configuration across all tested NKCC1 levels.

A final shared feature across both stoichiometries was the emergence of plateau regions in the learning landscape. In these regions, further increases in KCC2 activity at moderate NKCC1 levels had little impact on learning speed, as measured by the number of epochs required for convergence. These results highlight how non‐synaptic ionic mechanisms, shaped by specific biophysical properties such as cotransporter stoichiometry, can exert a profound influence on the computational capabilities of spiking neural networks.

### 3.1 Robustness to Initial Conditions

To ensure that the learning landscapes reported in this dissertation are not artifacts of a single network initialization, the complete 21 × 21 grid of (*S*_*KCC*2_, *S*_*NKCC*1_) combinations was simulated 10 times. Two independent sources of variability were introduced at the start of each repetition:

1. Membrane Potentials: To introduce variability in intrinsic excitability, a perturbation δ*V* ∈ [−1.5, +1.5] mV was added to the half‐activation voltage *V*_1/2_ of the gating variables associated with the voltage‐gated Na^+^ and K^+^ channels. Specifically, this shift was applied to *V*_1/2_ in the activation and inactivation functions (*m*, *h* for Na^+^; *n* for K^+^). Here, *V*_1/2_ denotes the membrane potential at which each gating variable equals 0.5, meaning that 50% of the corresponding gates are open (or not inactivated, in the case of *h*). This manipulation introduces diversity in the channels' voltage sensitivity, effectively varying the neurons' firing thresholds and thus their intrinsic excitability.
2. Synaptic weights: All initial synaptic weights were drawn from a uniform distribution *U*(0, 0.6) instead of the default fixed value of 0.3.

**FIGURE 5:**
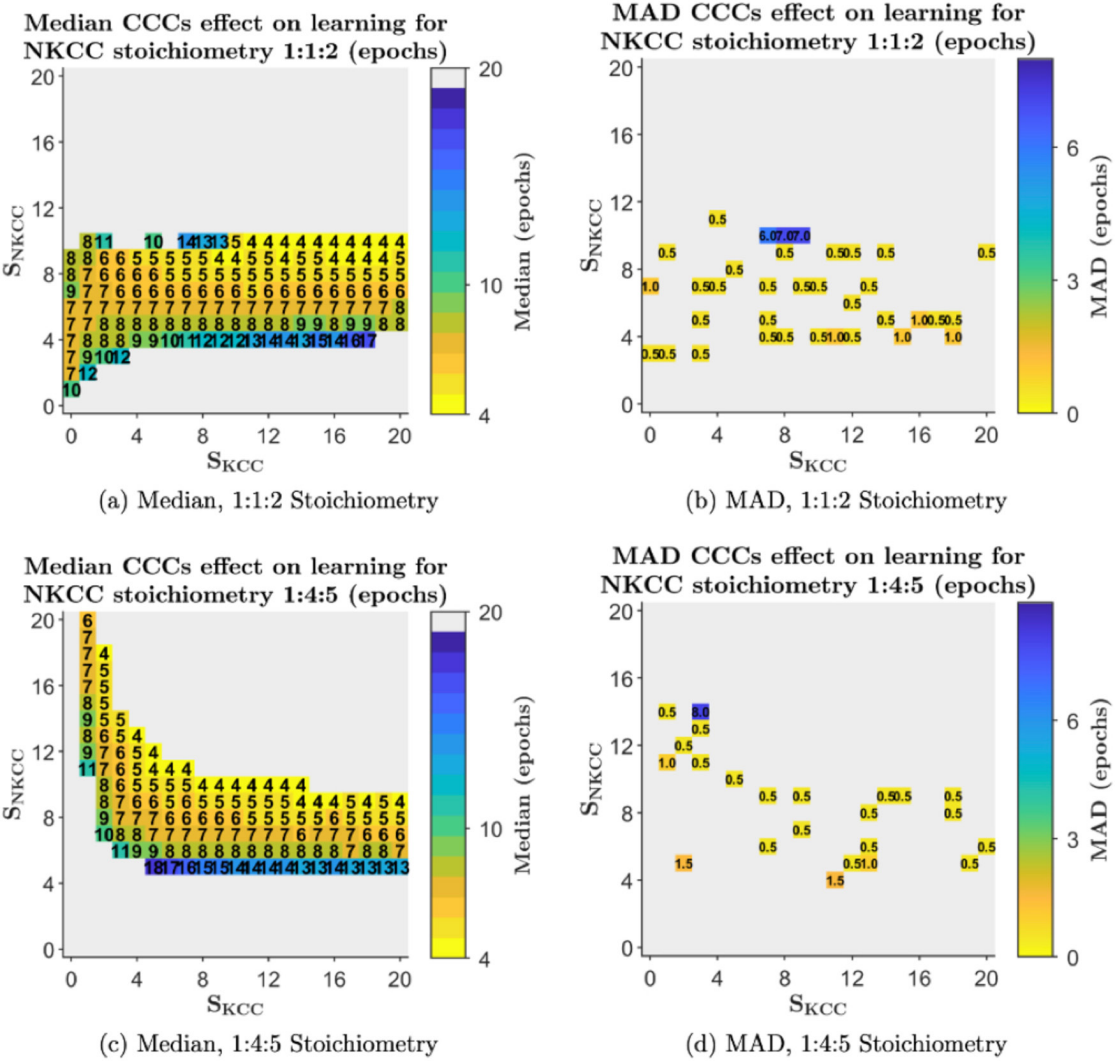
Robustness of the learning landscapes to variability in initial conditions. Each panel summarizes 10 full‐grid repetitions in which synaptic weights were redrawn from *U*(0, 0.6) and resting potentials were perturbed by ±1.5 mV. (A, B) NKCC1 stoichiometry 1:1:2; (C, D) stoichiometry 1:4:5. Left‐hand maps (A, C) give the median epochs required for convergence, reproducing the learning windows seen in Figure 4. Right‐hand maps (B, D) show the median absolute deviation (MAD) of the same epoch counts; values of 0–0.5 in the principal learning regions demonstrate that convergence speed remains highly consistent despite the randomized initial synaptic and intrinsic states.

For each (*S*_*KCC*2_, *S*_*NKCC*1_) pair, the median and MAD of the epochs‐to‐convergence were computed across the 10 repetitions. Figure 5 collects the resulting statistics.

The median number of epochs to convergence (Figure 5A for the 1:4:5 model, Figure 5C for the 1:1:2 model) reproduces the single‐run landscapes almost exactly (compared with Figure 4B,D). To quantitatively validate the similarity between the median convergence maps and the corresponding single‐run landscapes, we computed correlation‐based similarity measures across the full parameter grids (441 cells per map). For the NKCC1 stoichiometry 1:1:2, the median convergence map was strongly correlated with the single‐run landscape (Pearson's *r* = 0.7515, *p* = 2.6 × 10^−81^; Spearman's *ρ* = 0.7488, *p* = 1.9 × 10^−80^). An even higher agreement was observed for the 1:4:5 stoichiometry (Pearson's *r* = 0.8603, *p* = 1.6 × 10^−130^; Spearman's *ρ* = 0.8603, *p* = 1.5 × 10^−130^). The tapering learning band that characterizes the 1:4:5 stoichiometry and the broader, initially expanding plateau of the 1:1:2 stoichiometry are both preserved after the additional membrane‐potential noise.

Consistency across repetitions was quantified with the MAD (Figure 5B,D). In the main learning windows, MAD values remain at 0 or 0.5 epochs for both stoichiometries, confirming that convergence speed is essentially unchanged by the random initial conditions. Only a small cluster near (*S*_*KCC*2_ ≈ 7, *S*_*NKCC*1_ ≈ 8) in the 1:1:2 map shows a higher scatter (MAD ≈ 6–7 epochs), coinciding with the sharp edge of that model's learning plateau and therefore reflecting the expected sensitivity at a bifurcation boundary. Outside this narrow zone the variability never exceeds 1 epoch.

Taken together, these results show that the distinctive, stoichiometry‐dependent learning landscapes—and the narrow bands of fast convergence—are robust features of the network dynamics, not artifacts of a particular synaptic or intrinsic initialization. To assess whether the reported variability measures were sensitive to the number of repetitions, we evaluated the stability of the learning landscape and of the MAD as repetitions accumulated. For representative regions within and near the boundaries of the learning‐competent window, the median epochs‐to‐convergence and corresponding MAD values were monitored as a function of the number of repetitions. We observed that both metrics stabilized rapidly, with negligible changes beyond approximately 8–10 repetitions, and no qualitative alteration of the learning landscape topology. In particular, increasing the number of repetitions did not expand or contract the functional learning window, nor did it materially affect the MAD values within the principal convergence regions. These observations indicate that 10 repetitions per $\left(S_{\mathrm{KCC}2},S_{\text{NKCC}1}\right)$ pair are sufficient to capture the intrinsic variability induced by random initial conditions and that the robustness patterns reported in Figure 5 are not an artifact of under‐sampling.

## 4 Discussion

Our simulations demonstrate that the capacity for effective learning in a biophysical spiking network is gated by the combined activity of KCC2 and NKCC1. We found that successful learning is restricted to a contiguous “functional window” in the *S*_*KCC*2_ *–S*_*NKCC*1_ parameter space. Outside this window, learning consistently fails due to network states unsuitable for synaptic plasticity, which arise from either insufficient excitability preventing signal propagation or excessive excitability that saturates the network response. This finding provides a mechanistic basis for the principle that cognitive function requires a precise tuning of network excitability. Rather than being a static property, we show that this state is actively maintained by the dynamic interplay of opposing ion transport mechanisms that regulate intracellular chloride homeostasis.

An important question is whether the observed dependence of learning on KCC2/NKCC1 activity reflects a mechanism specific to chloride regulation or merely a generic tuning of network excitability. In the present model, cotransporter activity does not act as a static gain control but dynamically regulates intracellular chloride and extracellular potassium concentrations, thereby reshaping the polarity, efficacy, and temporal impact of GABAergic inhibition during learning. This dynamic coupling introduces feedback between ongoing activity, ionic gradients, and synaptic plasticity that cannot be reproduced by fixed manipulations such as clamping the inhibitory reversal potential or uniformly scaling excitatory or inhibitory conductances. Consistent with this distinction, learning performance does not vary monotonically with transporter activity, and networks with similar baseline firing levels can occupy qualitatively different learning regimes depending on chloride homeostasis. These features indicate that the reported learning landscapes reflect regime transitions imposed by ionic regulation rather than simple excitability shifts. While future work could further disentangle these mechanisms using explicit control simulations, the present results demonstrate that dynamically regulated chloride homeostasis constitutes a distinct and powerful constraint on the network states that support learning.

A potential concern is whether the qualitative differences observed between the 1 Na^+^:1 K^+^:2 Cl^−^ and 1 Na^+^:4 K^+^:5 Cl^−^ learning landscapes arise from the underlying stoichiometry or from the rescaling of the maximal flux coefficients (*Q*_*KCC*2_, *Q*_*NKCC*1_). The adjustment of these coefficients was a deliberate methodological step required to ensure that, for each stoichiometry, the explored parameter space spans a physiologically meaningful dynamic range of chloride regulation. Because different ion‐coupling ratios inherently imply different transport efficiencies and thermodynamic driving forces, using identical *Q* values across stoichiometries would collapse the system into trivially hyper‐ or hypo‐excitable regimes, preventing a fair comparison of learning dynamics. Importantly, the comparison between stoichiometries is therefore not based on absolute flux magnitudes, but on their effects within comparable functional regimes, where intracellular chloride concentration and chloride reversal potential vary over ranges consistent with effective inhibitory signaling. Within these normalized regimes, the two stoichiometries nevertheless generate qualitatively distinct learning landscapes, including differences in topology, width, and non‐monotonic dependence on transporter balance. This indicates that the observed effects are not artifacts of parameter tuning but emerge from fundamental differences in ion coupling, which shape how chloride and potassium fluxes interact with network dynamics. Future extensions could further formalize this comparison by explicitly matching baseline *E*_Cl_ ranges across stoichiometries; however, the present results already demonstrate that stoichiometry imposes intrinsic constraints on learning that persist across equivalent functional operating regimes.

A direct quantitative comparison between the two NKCC1 stoichiometries was performed at the level of learning landscapes rather than by pointwise statistical testing of individual parameter combinations. For each stoichiometry, learning performance was summarized across repetitions using the median number of epochs to convergence and the corresponding MAD, as reported in Figure 5. This approach captures both central tendency and variability in a manner robust to the highly non‐Gaussian distributions that arise near the boundaries of the learning‐competent window. Importantly, the two stoichiometries differ not only in average convergence speed but also, and more fundamentally, in the topology of the learning landscape: the extent, shape, and continuity of the parameter regions supporting successful learning. Quantitative differences are therefore reflected in measurable global features, such as the area of the parameter space that supports convergence, the width of the learning band along the $S_{\mathrm{KCC}2}$ and $S_{\text{NKCC}1}$ axes, and the sharpness of the transition between convergent and non‐convergent regimes. These landscape‐level differences are preserved across repetitions and are evident in both the median and MAD maps (Figure 5), indicating that the effects of stoichiometry on learning dynamics are robust and not attributable to stochastic variability. While classical statistical comparisons (e.g., mean ± SEM at fixed parameter points) are less informative in this context due to threshold‐like behavior and regime transitions, the consistency of these global quantitative features provides strong evidence that learning dynamics differ systematically between the 1:1:2 and 1:4:5 stoichiometries.

Beyond the existence of this functional window, our results show that its shape and characteristics are qualitatively transformed by the operational stoichiometry of NKCC1. With the canonical 1:1:2 stoichiometry, increasing *S*_*KCC*2_ acts as a straightforward stabilizer, widening the range of tolerable *S*_*NKCC*1_ levels, as would be expected from KCC2's classical hyperpolarizing action on ECl (Figure 4D). In contrast, the thermodynamically favored 1:4:5 stoichiometry—proposed for immature neurons (Brumback and Staley 2008)—produces a more complex learning landscape where the functional window forms a tapering band. This band achieves its maximum width, representing the most permissive range of *S*_*NKCC*1_ activity, in the region of low *S*_*KCC*2_. This specific configuration faithfully mirrors the transporter expression patterns characteristic of the neonatal brain (Peerboom and Wierenga 2021; Kahle, Staley, Nahed, Barrett, et al. 2008).

The mechanisms driving this complex, stoichiometry‐dependent landscape are further illuminated by the network's response to a complete KCC2 blockade. A striking effect emerged when *S*_*KCC*2_ was set to zero: learning persisted in the 1:1:2 model but collapsed entirely in the 1:4:5 model, even though baseline network activity and signal propagation remained viable. This dichotomy highlights that KCC2's role in the 1:4:5 model transcends simple excitability tuning. This sensitivity can be understood through the dual action of KCC2 transport. While its primary effect is to extrude Cl^−^ and stabilize the neuron, the coupled efflux of K^+^, under conditions of high transporter flux, can lead to the accumulation of extracellular potassium ([K^+^]_*o*_) (Thompson and Gähwiler 1989), which depolarizes the membrane and can even reverse the KCC2 driving force (Russell 2000a; Blaesse et al. 2009). This dual mechanism offers a potential explanation for the non‐monotonic learning efficiency observed in the 1:4:5 landscape. One interpretation is that this finding provides computational support for non‐canonical, plasticity‐related functions of KCC2 reported experimentally (Li et al. 2007; Chevy et al. 2015; Gauvain et al. 2011). An alternative interpretation is that the network failure in this high‐flux regime reflects the absence of other homeostatic processes in our model, most notably glial potassium buffering, which would mitigate such runaway excitability in vivo. Future work could investigate the contribution of glial cells to chloride homeostasis, including their role in buffering extracellular ions and dynamically modulating neuronal chloride gradients, which are not explicitly captured in the current model.

A common feature in both learning landscapes was the saturation of learning efficiency. Beyond a certain threshold, particularly at moderate *S*_*NKCC*1_ levels, further increases in *S*_*KCC*2_ did not substantially improve learning speed. Instead, performance remained robust across a wide range of *S*_*KCC*2_ values, until a critical tipping point was reached where excessive hyperpolarization prevented effective signal propagation to the output neuron. This saturation embodies the principle of biological robustness, where complex systems are often organized to buffer their function against significant variation in underlying parameters (Kitano 2004; Marder and Goaillard 2006). From a practical standpoint, this finding suggests that therapeutic or experimental manipulations targeting KCC2 may operate within a relatively broad range of expression levels in which moderate overexpression does not degrade network‐level performance within the present modeling framework. This interpretation should nevertheless be treated with caution, as biological tissues are highly plastic and may adapt to altered transporter configurations through compensatory mechanisms.

Previous computational studies have investigated the role of CCCs in regulating neuronal excitability. For instance, network‐level models have demonstrated that dysregulation of KCC2 or NKCC1 can promote the emergence of seizure‐like dynamics (Buchin et al. 2016; De Almeida et al. 2012). At the single‐cell level, other models have shown how activity‐dependent chloride accumulation alters neuronal input–output properties (Currin et al. 2020), degrades the precision of spike timing (Doyon et al. 2016), and interacts with neuronal morphology to determine the efficacy of inhibition (Currin and Raimondo 2022; Jedlicka et al. 2011). Development‐focused simulations have also confirmed that NKCC1‐mediated transport is sufficient to maintain chloride homeostasis in immature neurons (Kolbaev et al. 2020). While these studies provide essential insights into how CCC activity modulates excitability and ion dynamics, the functional consequences of the combined KCC2–NKCC1 interplay for network‐level learning have remained largely unexplored.

Our model is also distinguished from more abstract frameworks of network learning. Classical rate‐based models (Hopfield 1982) and many contemporary spiking models that implement Hebbian or spike‐timing‐dependent plasticity (Song et al. 2000; Vogels et al. 2011) typically treat neuronal excitability as a static parameter. Consequently, they cannot capture the crucial feedback loop in which learning itself perturbs ionic gradients, which in turn influences subsequent plasticity. By contrast, our network couples neurons through a discrete Hebbian/anti‐Hebbian, ionic diffusion, and dynamic CCC kinetics. This allows transporter‐mediated shifts in ECl and [K^+^]_*o*_ to influence—and be influenced by—plasticity on every time‐step. In this way, our model integrates the explicit ionic bookkeeping often absent from high‐performance surrogate‐gradient frameworks (Zenke and Vogels 2021) into a microcircuit that still performs a well‐defined learning task. This provides a bridge between efficient SNN training techniques and the principles of experimentally testable, energy‐constrained biophysics.

The biophysically detailed network model employed in this study is also distinguished from more phenomenological frameworks of network learning. The inclusion of the GHK model of neuronal membrane potential, dynamics of ion concentrations and thus effect of electrodiffusion provides the necessary infrastructure to investigate the role of subcellular elements on learning. This level of mechanistic detail is what allows the model to faithfully translate the effects of KCC2 and NKCC1 activity on intracellular chloride homeostasis into a measurable impact on the network's learning performance. In contrast, while many contemporary spiking models can effectively reproduce complex firing patterns (Song et al. 2000; Vogels et al. 2011), they are phenomenological in nature. They typically represent excitability as a set of fixed parameters and lack the underlying ionic machinery to simulate the effects of transporter activity. Consequently, these models cannot be used to explore the feedback loop between ion regulation and synaptic plasticity. The approach used here, therefore, leverages the mechanistic depth of the network model to bridge the gap between the cellular biophysics of ion transport and the emergent, cognitive function of network‐level learning.

However, our model has some limitations. If it incorporates crucial non‐synaptic mechanisms including ionic electrodiffusion and dynamic volume changes, the neurons are solely modeled as single compartments. This point‐neuron representation precludes an investigation of how dendritic morphology might interact with localized ion accumulation and transporter distribution to shape learning. For instance, spatially distinct dynamics within the dendritic tree could introduce additional complexities not captured here. Furthermore, the absence of glial cells means that important homeostatic processes, particularly astrocytic potassium buffering, are not accounted for; incorporating this would likely sharpen the observed saturation thresholds. Nevertheless, the model generates clear, experimentally testable predictions. The non‐monotonic dependence of learning on KCC2 activity in the 1:4:5‐like regime, for example, could be probed by using chemogenetic tools to precisely titrate KCC2 expression levels in relevant cortical circuits during a learning task.

To relate the model parameters to experimentally established regimes of chloride regulation, it is important to emphasize that the activity scalers *S*_*KCC*2_ and *S*_*NKCC*1_ are dimensionless quantities that capture relative transporter efficacy rather than absolute expression levels or single‐molecule fluxes. Within this framework, the learning landscapes identified here can nevertheless be interpreted in relation to experimentally characterized regimes of chloride homeostasis. During early development, elevated NKCC1 activity combined with low KCC2 expression sustains high intracellular chloride concentrations and depolarizing GABAergic signaling. This condition is favorable for the early postnatal development, facilitating the maturation of synapses—excitatory and inhibitory (Pfeffer et al. 2009). In the present work, this condition was represented by the DP. The progressive upregulation of KCC2 during the postnatal GABA shift lowers intracellular chloride and stabilizes inhibitory efficacy (Peerboom and Wierenga 2021; Kaila et al. 2014; Rivera et al. 1999). In the model, this transition corresponds to movement across the *S*_*KCC*2_–*S*_*NKCC*1_ parameter space toward a bounded functional region in which learning is robust. Notably, experimental studies indicate that mature neurons operate within a relatively narrow range of chloride reversal potentials compatible with effective inhibition, a constraint that is qualitatively mirrored by the sharp learning boundaries observed in the simulations (Kaila et al. 2014). Conversely, pathological conditions associated with cognitive impairment—such as epilepsy, neurodevelopmental disorders, and schizophrenia—often involve reduced KCC2 function and/or persistent NKCC1 activity, leading to weakened or depolarizing inhibition. In the context of the present model, such alterations map onto parameter regimes outside the learning‐competent region, offering a mechanistic interpretation for experimentally reported learning deficits and their partial rescue following pharmacological or genetic modulation of cation–chloride cotransporters. While the model is not intended to quantitatively reproduce a specific experimental preparation, it captures how experimentally observed shifts in transporter balance can constrain the network states that support learning.

In a broader context, our model provides a quantitative map of the challenges a network faces during developmental transitions like the GABA shift. It illustrates a narrow functional tolerance, where small changes in transporter activity can shift the network from a functional to a non‐functional state. Ultimately, this work provides a clear answer to its principal question. We conclude that KCC2 and NKCC1 act as critical computational modulators of learning, creating a complex landscape of network states where plasticity is viable. Their influence extends beyond merely setting a homeostatic excitation‐inhibition balance. Instead, the key determinant of a network's capacity for effective learning is the specific, dynamic interplay between their activities, an interaction governed by fundamental biophysical properties like stoichiometry. By linking ionic homeostasis to learning efficiency, this work points toward a new class of interventions that target CCCs—not merely to correct excitability disorders, but to modulate the cellular conditions that enable effective learning.

## 5 Conclusion

This study provides a quantitative link between the regulation of chloride homeostasis by KCC2 and NKCC1 and the learning capacity of a biophysical spiking neural network. Our simulations demonstrate that efficient learning is confined to a functional window of transporter activities, the characteristics of which are qualitatively transformed by the transporter's ion coupling stoichiometry. We conclude that the specific, dynamic interplay between these non‐synaptic ion transporters, governed by fundamental biophysical properties, is a key determinant of a network's ability to perform a cognitive task. This work suggests that such mechanisms do not merely set a homeostatic background for computation, but are themselves active and powerful modulators of learning.

## Author Contributions

**Mohamed Ahmed:** data curation, formal analysis, investigation, validation, visualization, writing – original draft, writing – review and editing. **Antônio‐Carlos Guimarães de Almeida:** conceptualization, formal analysis, funding acquisition, investigation, methodology, resources, supervision, validation, visualization, writing – review and editing. **Antônio Márcio Rodrigues:** conceptualization, formal analysis, investigation, methodology, resources, software, supervision, validation, visualization, writing – review and editing. **Damien Depannemaecker:** conceptualization, investigation, methodology, software, supervision, validation, visualization, writing – review and editing.

## Conflicts of Interest

The authors declare no conflicts of interest.

## Data Availability

Data and model are available upon request.
